# Local inhomogeneous state in multiferroic SmCrO_3_

**DOI:** 10.1038/s41598-020-61384-6

**Published:** 2020-03-13

**Authors:** G. N. P. Oliveira, R. C. Teixeira, R. P. Moreira, J. G. Correia, J. P. Araújo, A. M. L. Lopes

**Affiliations:** 1 IFIMUP-Instituto de Física de Materiais Avançados, Nanotecnologia e Fotónica, Departamento de Física e Astronomia da Faculdade de Ciências da Universidade do Porto, Rua do Campo Alegre, 687, 4169-007 Porto, Portugal; 2 C2TN, Centro de Ciências e Tecnologias Nucleares, Departamento de Engenharia e Ciências Nucleares, Instituto Superior Técnico, Universidade de Lisboa, Estrada Nacional 10, 2695-066 Bobadela, LRS Portugal

**Keywords:** Physics, Condensed-matter physics, Ferroelectrics and multiferroics

## Abstract

Rare-earth orthochromites with distorted perovskite structure (e.g. RCrO_3_, R = Sm, Gd) have been under strong debate with respect to the origin of their ferroelectric order. Of particular interest is the question of whether such orthochromites are, in fact, magnetically driven improper ferroelectrics, as many rare-earth manganites or orthoferrites. Here we show, by studying at the atomic scale the rare-earth SmCrO_3_ system that a distortion of the Sm local environment emerges within the paramagnetic phase, near room temperature. Our Electric Field Gradient measurements combined with first-principles calculations show that the emergent phase cannot be simply ascribed to the *Pna*2_1_ structure as reported for GdCrO_3_ or SmCrO_3_. Instead a local inhomogeneous state, where regular non-polar and polar distorted environments coexist, develops at low temperatures.

## Introduction

Almost a decade ago, it was found that rare-earth orthochromites (perovskite-like oxides) with formula RCrO_3_ (*e.g*. R = Yb, Er, Y, Lu and Sm) exhibit a rich variety of physical properties, making them a fruitful playground for both fundamental materials physics and applied related studies. Unique attributes such as magnetisation reversal (or negative magnetisation) phenomena, exchange bias and magnetisation switching were recently observed in orthochromites^1–8^. The different ferroic orders that these multiferroic perovskite-like materials may exhibit, such as ferromagnetism, ferroelectricity, and/or ferroelasticity, further contribute to their scientific relevance^9–11^.

RCrO_3_ compounds, deviate from an ideal cubic structure, due to the ion’s sizes, crystallise in the orthorhombic GdFeO_3_-type perovskite-like structure (space group *Pnma*)^12,13^. There, the larger R atoms are located at the center of a cube, with 8 Cr atoms on the vertices and with the 12 O atoms occupying the middle of each cube’s edges. Different structural distortions are present in these compounds (from cubic to orthorhombic or rhomboedric symmetries)^14–18^, which can be driven by external parameters like temperature, pressure or chemical composition. In fact, it has been shown that induced distortions in this structure may lead to new magnetic and electrical properties^19,20^.

In such canted antiferromagnets with a weak ferromagnetic component, the Néel magnetic transition temperature (${T}_{\,{\rm{N}}}^{{\rm{Cr}}\,}$, Cr sub-lattice ordering temperature) decreases with decreasing radius of the R^3+^ ions (*e.g*., *T*_N_ = 288 K in LaCrO_3_ and *T*_N_ = 112 K in LuCrO_3_). Some RCrO_3_ compounds with R = Nd, Sm, Gd, Er show Cr^3+^ spin-reorientation transitions at lower temperatures^6,21^. At very low temperatures the spins of R^3+^ ions undergo antiferromagnetic (AFM) ordering (${T}_{\,{\rm{N}}}^{{\rm{R}}\,}$).

Recently, rare-earth orthochromites with distorted perovskite structure, especially GdCrO_3_, SmCrO_3_ and YCrO_3_ have been the focus of great interest. Ferroelectric (FE) behaviour was experimentally reported to arise just above the antiferromagnetic ordering temperature *T*_N_ leading to a debate concerning their low temperature structural phase. Indeed, the correct structural assignment is a challenging task as long-range average methods are here insufficient to clearly distinguish among very similar structures^22–25^. Also, controversy persists regarding these materials’ ferroelectricity, its true existence and origin^22–24,26,27^. A recent theoretical investigation debates the sources of improper electric polarisation in rare-earth orthoferrite and orthochromite perovskites^28^ and claims that a magnetostructural coupling underlies the ferroelectricity observed in these materials suggesting that these systems are magnetically driven improper ferroelectrics, challenging the previous experimental observations^22–25^.

Early works report dielectric permittivity anomalies near 400–500 K in rare-earth chromites associated with non-centrosymmetry^12^. Other authors^20^ claim that the observed polarisation is driven by magnetic order due to the combined effect of the poling-field breaking the R ion local symmetry and the exchange-field due to the weak ferromagnetic component of the Cr sub-lattice. A strong spin-phonon correlation along the displacement of the R ion has also been proposed to explain Raman spectroscopy results^29^. According to these works, no spontaneous ferroelectric polar-order exists, and the presence of a magnetic R-ion is essential to induce a metastable ferroelectric state. The appearance of ferroelectricity near room temperature, arising from polar octahedral rotations and/or off-center cation displacements was recently claimed^22,23,26,27^ having no correlation with the magnetic order. Additionally, it has been recently argued that in some RCrO3 perovskites (*e.g*. R = Ho, Gd and Sm) ferroelectricity is due to a symmetry change from the high temperature *Pnma* to the low temperature non-centrosymmetric *Pna*2_1_ phase^24,26,30–32^. Furthermore, *Serrao et al*. using first-principles density functional theory calculations, suggested that in the case of YCrO_3_, a structural phase transition also occurs to the monoclinic *P*2_1_ (n°4), although they believe that a small distortion of the structure could amount to a further lowering of the symmetry^24,25,33^. Claims for ferroelectricity exist for cases where R = Dy, Ho, Yb, Er, Y, Lu, Nd, Sm but not when R = La or Pr^12,13,33–35^.

Although these systems have been claimed as potential magnetoelectric materials additional efforts are necessary for providing unambiguous confirmation. In fact, ferroelectricity in RCrO_3_ (R = Ho-Lu and Y) has not yet been demonstrated in contrast to the La_1−*x*_Bi_*x*_CrO_3_ system where ferroelectric hysteresis loops were observed^36^. Furthermore, *Lebedev et al*. reported that both LuCrO_3_ and ErCrO_3_ show the presence of ferroelectric-like properties, with the latter having a much stronger magneto-dielectric effect, as expected, due to the different R-site cation and its distinct magnetic properties^1^. In SmCrO_3_, a member of the RCrO_3_ series, the easy magnetic axis and the origin of ferroelectric and magnetoelectric effects are still under debate. Synchrotron diffraction studies performed on SmCrO_3_, suggest that the appearance of FE order about 27 K above *T*_N_ is due to a structural distortion occurring at 240 K, concomitantly with the onset of the polar order^22^.

The assessment of new materials for new applications relies on the understanding and control of their fundamental properties^37,38^. Since these properties might arise from local features that are not well studied nor described by methods based on long-range average structural properties, the use of local probe studies is essential^6,12,16,22,39^. In this context, hyperfine methods, such as perturbed angular correlation (PAC) spectroscopy, might provide relevant nanoscale information on magnetic and electric local structural properties of these perovskite-like systems. In particular, the electric field gradient (EFG) interacting with a certain probe nuclei provides the signature of its local environment. Revealing the density and asymmetry of the local charge distribution, the EFG allows one to infer about the atomic and electronic environment of the probe nuclei. PAC has given proof of being a valuable technique to study local anomalies in complex oxides^37,40–42^ that we now apply to study SmCrO_3_.

In this work SmCrO_3_ samples were studied using the PAC technique over a temperature range comprehending the relevant magnetic and (suggested) ferroelectric phase transitions.

## Results and Discussion

### Structural and magnetic characterisation

**Figure 1:**
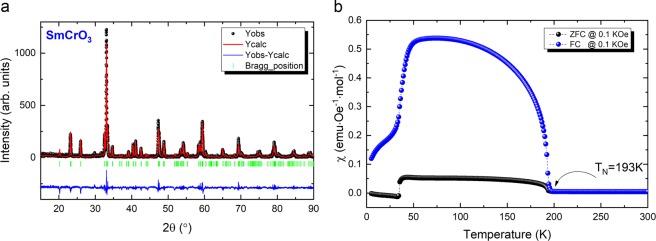
SmCrO_3_ structural and magnetic characterisation results. (**a**) Room temperature powder XRD pattern of SmCrO_3_ sample after the final heat treatment. Experimental pattern (dots), fit curve (red line) and residual difference (blue line). *Pnma* phase Bragg reflections (vertical bars). (**b**) Isofield [M(T)] curves (ZFC and FC) for SmCrO_3_ taken with an applied field of 0.1 kOe.

 Figure 1a presents the graphical output of the Rietveld refinement performed to the acquired XRD pattern of the SmCrO_3_ sample after the final heat treatment.

All the diffraction peaks were indexed within the orthorhombic perovskite structure (single phase pattern) that belongs to the space group *Pnma*. The obtained values (*a* = 5.4970, *b* = 7.6439 and *c* = 5.3675) are in good agreement with those reported in the literature evidencing the good quality of the sample^12^. The values obtained in the last refinement cycle are summarised in Table 1.

**Table 1:** Structural parameters and atomic positions for SmCrO_3_ system at room temperature.

**SG**:*Pnma*	*a* (Å) 5.4970(4)	*b* (Å) 7.6439(5)	*c* (Å) 5.3675(2)	*U*_*iso*_(Å^2^)	*Occ*
Atom	*x*	*y*	*z*		
Sm: 4*c*(*x*, 0.25, *z*)	0.0543	0.2500	− 0.0135	0.577	0.549
Cr: 4*b*(0, 0, 0.5)	0	0	0.5	0.567	0.576
O(1): 4*c*(*x*, 0.25, *z*)	0.4816	0.25	0.0923	0.790	0.700
O(2): 8*d*(*x*, *y*, *z*)	0.2909	0.0451	− 0.2964	1.449	1.849
Cr-O1-Cr = 146.9°	***R***_***wp***_ = 11.1	***R***_*p*_ = 8.38	***R***_*exp*_ = 9.07	***χ***^2^ = 1.65	

 Figure 1b presents an isofield [M(T)] curve for SmCrO_3_ sample taken with an applied field of 0.1 kOe. The system presents the expected characteristics, whereby the Cr^3+^ spins order antiferromagnetically below *T*_N_ = 193 K, undergoing a reorientation at 34 K and the second magnetic sub-lattice (Sm) orders at 20 K. A weak ferromagnetic moment also arises due to the canting of the Cr atoms (for further details on the crystallographic and magnetic characterisation see ref. ^18^).

### Perturbed angular correlation experiments

PAC experiments on the distorted perovskite SmCrO_3_ sample were performed both by diffusing the sample with ^111^In and by implanting it with ^111*m*^Cd. The EFG parameters were probed as a function of temperature, *T*, within the 723 K > *T* > 16 K interval that spans over reportedly relevant transition temperatures: ferroelectric transition (*T*_FE_ = 220 K), the magnetic ordering of the Cr sublattice (${T}_{\,{\rm{N}}}^{{\rm{Cr}}\,}=193$ K), the spin reorientation (*T*_SR_ = 34 K) and the magnetic ordering of the Sm sublattice (${T}_{\,{\rm{N}}}^{{\rm{Sm}}\,}=20$ K).

**Figure 2:**
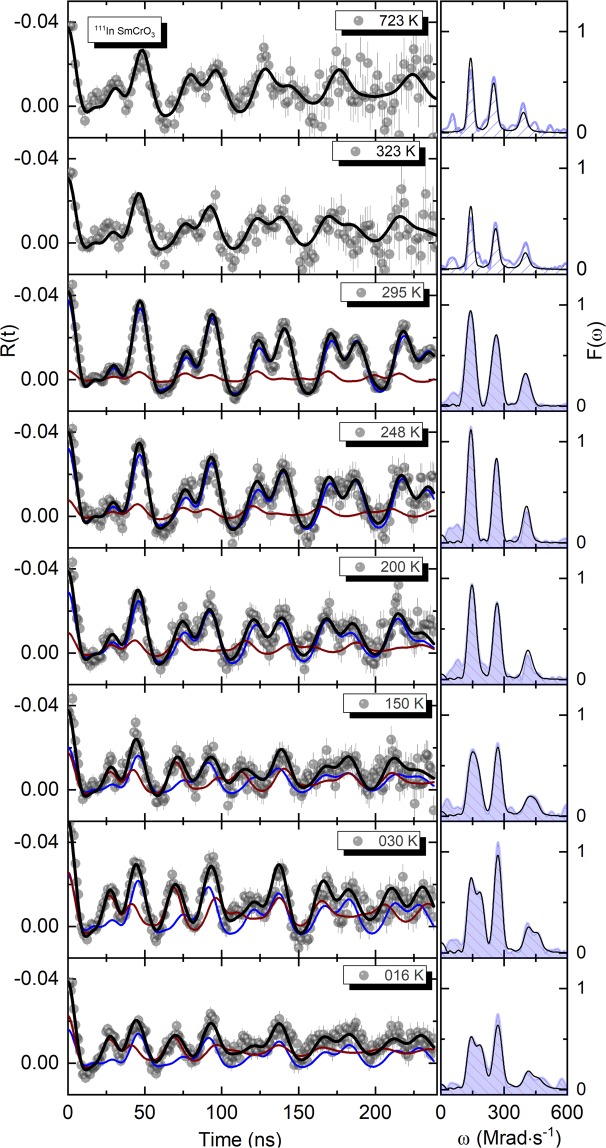
Representative *R*(*t*) functions, corresponding fits and respective Fourier transform (black lines) taken at different temperatures for the ^111^In parent probes in SmCrO_3_. Blue and brick color lines show EFG_1_ and EFG_2_ contributions, repectively.

 Figure 2 depicts the experimental *R*(*t*) anisotropy function (left) together with the Fourier transforms [Fts] (right). The global fits to the *R*(*t*) functions are shown by the thick continuous black lines in the spectra.

At high temperatures, above 300 K, a simple visual inspection shows that there are no significant changes in the spectra. Here, a single frequency triplet is clearly observable *i.e*., evidencing one single probe local environment. Accordingly, the fits to the *R*(*t*) experimental data were performed considering only one static regular EFG_1_ distribution characterised by the fundamental frequency ${\omega }_{0}^{{\rm{EF}}{{\rm{G}}}_{1}}=132(2)\ {\rm{Mrad}}$/${\rm{s}}({V}_{zz}^{{\rm{E}}{\rm{F}}{{\rm{G}}}_{1}}=76(3){\rm{V}}/{\mathring{\rm A} }^{2})$ and asymmetry parameter *η* = 0.23(3) (indicative of a slight axial asymmetry of the charge distribution), with a slight attenuation described by a Lorentzian function with a relative FWHM < 1%.

A very interesting aspect of our SmCrO_3_ PAC results is revealed for temperatures below 300 K, where visible changes can be observed in the perturbation function *R*(*t*) and corresponding Fts. In detail, a second distribution, EFG_2_ emerges and its relative abundance increases with decreasing temperature. Thus, the fits to the *R*(*t*) experimental data were performed considering two EFG (EFG_1_ and EFG_2_) distributions. EFG_2_ is characterised by a similar fundamental frequency, ${\omega }_{0}^{{\rm{EF}}{{\rm{G}}}_{2}}=132(3)\ {\rm{Mrad}}/{\rm{s}}$ (${V}_{zz}^{{\rm{E}}{\rm{F}}{{\rm{G}}}_{2}}=75(5){\rm{V}}/{\mathring{\rm A} }^{2}$) and an asymmetry parameter slightly higher *η* = 0.37(5) than EFG_1_.

### Density functional theory simulations

In order to extract maximum information from the PAC experimental results, *i.e*. understanding the probe’s location and local environment, *ab-initio* calculations of the hyperfine parameters at the R and Cr sites, on the orthorhombic lattice of the RCrO_3_ family, were performed. Density functional theory calculations were carried out using the linearised augmented plane wave+local orbitals method (LAPW+lo) as implemented in the WIEN2k code^43,44^. The calculations were performed considering pure orthorhombic compounds using a set of lattice parameters taken from the literature^45^, to have the best precision on the atomic positions. The relaxation of internal atomic positions was allowed (minimising the atomic forces to values less than 5 mRy/a.u.). The calculation halted when the difference charge, energy and force were less than 0.001 e, 0.0001 Ry and 1 mRy/a.u., respectively for all compounds. The muffin-tin radii *R*_*MT*_ for each compound are summarised in Table 2.

**Table 2:** Muffin-tin radius *R*_*MT*_ for the RCrO_3_ (R = Yb, Er, Y, Sm, Gd, Nd, La) compounds.

Sample	***R***_***MT***_(**R**) (a.u.)	***R***_***MT***_(**Cr**) (a.u.)	***R***_***MT***_(**O**) (a.u.)
YbCrO_3_	2.23	1.89	1.71
ErCrO_3_	2.24	1.90	1.72
YCrO_3_	2.16	1.91	1.72
SmCrO_3_	2.32	1.89	1.71
GdCrO_3_	2.27	1.89	1.71
NdCrO_3_	2.31	1.88	1.70
LaCrO_3_	2.38	1.85	1.68

The cutoff energy, which defines the separation between valence and core states was chosen to be −6 Ry. Inside the atomic spheres, the partial waves were expanded up to *l*_*max*_ = 10. Several *k*-point grids and maximum wave numbers for the plane waves were tested in order to reach a good convergence of the *V*_*zz*_ and *η* parameters. In this way, the number of plane waves was limited by a cutoff *R*_*MT*_*K*_*max*_ = 7.5 and a *k*-mesh of 120 K-points in the irreducible Brillouin zone was used.

The exchange correlation potential was calculated using a Perdew-Becke-Erzenhof generalized gradient approximation (PBE)-GGA^46^ following the work of *Ong et al*.^47^.

A ferromagnetic configuration was considered for simplicity. This approach has proven to show good results for the calculation of EFG parameters^48,49^.

Due to computing time restrictions, full DFT calculations using large supercells where the actual Cd probe atom would be present as a highly diluted impurity could not be performed. Alternatively, a correction of the R and Cr lattice atoms EFGs was performed, inspired by old Point Charge Model (PCM) EFG calculations. Hence, the *V*_*zz*_ calculated by DFT for a host lattice atom at the R and Cr sites is scaled by the respective ratios of the Sternheimer anti-shielding factor (*γ*_∞_) of the probe ion Cd^2+^ by the corresponding value of the replaced lattice ion. This approximation simply assumes that the main differences between the EFG that a probe atom sees and the one seen by a lattice atom are due to electronic shells deformations induced by the external ionic and electronic lattice charge contributions. Although this does not in general lead to fully correct EFG values, it will provide the magnitude and trend for the expected EFGs interacting with the probe atom at the relevant different lattice sites.

**Figure 3:**
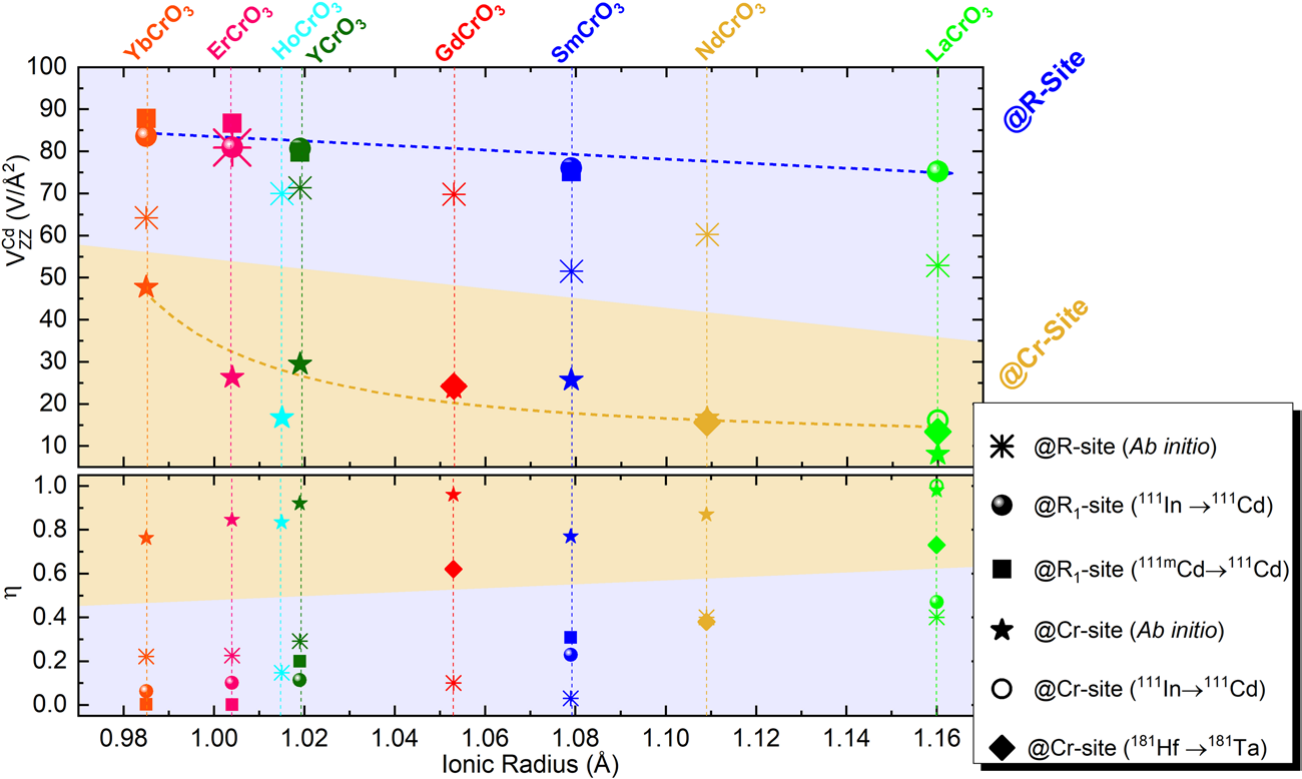
Linearised augmented plane wave + local orbitals method EFG parameters in the rare- earth and chromium sites for the orthorhombic RCrO_3_. Data on (Gd, Nd, La)CrO_3_ using ^181^Hf parent probe was taken from refs. ^50,51^. The dashed lines are guidelines to the eyes. Error bars are proportional to point size.

The EFG (*V*_*zz*_ and *η*) parameters estimated for a Cd probe at the R and Cr lattice sites are presented in Fig. 3 and summarised in Table 3. The corresponding experimental EFG values measured using the Cd probe obtained from the decay of ^111^In and ^111*m*^Cd parent probe isotopes together with data on (Gd, Nd, La)CrO_3_ using the ^181^Hf parent probe taken from literature are also presented^50,51^.

**Table 3:** EFG parameters in the rare- earth and chromium sites for the orthorhombic RCrO_3_. Data on (Gd, Nd, La)CrO_3_ using the ^181^Hf parent probe was taken from refs. ^50,51^.

Sample	R-site		Cr-site					
	***V***_***zz***_ (V/Å^2^)	*η* ( ± 0.05)	***V***_***zz***_ (V/Å^2^)	*η* ( ± 0.05)	Probe Cd/Ta^a^	Symbol		Refs.
YbCrO_3_	64.0(3)	0.22	48.0(3)	0.76	*ab-initio*^b^	✳	★	—
	84(2)	0.06			^111^In	●		—
	88(3)	0.00			^111*m*^Cd	■		—
ErCrO_3_	81.0(3)	0.22	26.0(3)	0.84	*ab-initio*	✳	★	—
	80(2)	0.10			^111^In	●		—
	87(3)	0.00			^111*m*^Cd	■		—
HoCrO_3_	70.0(3)	0.15	17.0(3)	0.83	*ab-initio*^b^	✳	★	—
YCrO_3_	71.0(3)	0.29	30.0(3)	0.92	*ab-initio*^b^	✳	★	—
	81(2)	0.11			^111^In	●		—
	80(3)	0.20			^111*m*^Cd	■		—
GdCrO_3_	70.0(3)	0.10	24.0(3)	0.96	*ab-initio*^b^	✳	★	—
			24(4)	0.62	^181^Hf		◆	^50,51^
**SmCrO**_**3**_	52.0(3)	0.03	26.0(3)	0.77	*ab-initio*^b^	✳	★	—
	76(2)	0.23			^111^In	●		—
	75(3)	0.31			^111*m*^Cd	■		—
NdCrO_3_	60.0(3)	0.40	17.0(3)	0.87	*ab-initio*^b^	✳	★	—
			15(4)	0.73	^181^Hf		◆	^50,51^
LaCrO_3_	53.0(3)	0.40	8.0(3)	0.98	*ab-initio*^b^	✳	★	—
	75(2)	0.47	16(4)	1.0	^111^In	●	○	^53^
			13(4)	0.73	^181^Hf		◆	^50,51^

^a^^111^In and ^111*m*^Cd isotopes decay to the same PAC probe nuclear state (Cd), while ^181^Hf decays to ^181^Ta. All PAC measurements are performed in the final, daughter state.

^b^*ab-initio* values are scaled by the respective Sternheimer anti-shielding ratio factors as explained in the text. A relative uncertainty of ~0.5% is attributed at the DFT estimated values due to the convergence criteria used in the calculation. The value is taken by overestimation.

### Probes location

The *V*_*zz*_ estimated at the rare-earth site of SmCrO_3_ (${V}_{zz}^{{\rm{S}}{\rm{m}}}=52.0(3){\rm{V}}/{\mathring{\rm A} }^{2}$) is about two times larger than the value obtained for Cd sitting at the Cr site (${V}_{zz}^{{\rm{C}}{\rm{r}}}=26.0(3){\rm{V}}/{\mathring{\rm A} }^{2}$). From the DFT data, lower values of *η* are expected due to a low distortion from axial symmetry at the R site, while larger values of *η* > 0.5 are expected at the Cr site, due to a lack of axial symmetry. Furthermore, there is a clear trend of these two parameters, as evidenced by the guidelines in Fig. 3, where *V*_*zz*_ decreases and *η* increases as a function of the R ionic radius, respectively. In fact, *Rearick et al*. observed in orthoferrites that in the heavier rare-earth systems, the R-site EFGs are nearly axially symmetric and, as the R atomic number decreases, the EFG asymmetry *η* increases^52^ while *V*_*zz*_ slowly decreases. Due to the fact that our experimental results (*V*_*zz*_ and *η*) and EFG estimations for Cd at the R site reasonably fit within the discussed trends for all compounds, we assume, that the experimental ${{\rm{EFG}}}^{{{\rm{R}}}_{1}}$ is related with Cd substituting the R site in RCrO_3_, in particular the Sm site in SmCrO_3_.

Regarding previous experiments where the ^111^In → ^111^Cd probe was introduced into LaCoO_3_ and La(Cr, Fe)O_3_ by a chemical process during synthesis, two different EFG interactions were observed and assigned to the two non-equivalent R- and Cr/Fe-sites, respectively. *Rearick et al*. reported that for the heavier rare-earths using the ^111^In parent probe, an In\Cd atom primarily substitute R-sites while in the lighter rare-earth compounds (larger ionic radius) In\Cd can substitute into both the Fe- and the R-sites^52^. Similar findings were obtained in LaCoO_3_ where only R-site substitution was observed by *Dogra et al*., while for LaCrO_3_ and (La, Lu, Ho, Eu, Y, Yb)FeO_3_ both sites were observed to be occupied by In\Cd^52,53^. In the present work, we do not observe an EFG that could be attributed to In\Cd probes sitting at the Cr site. We hint that this might be due to the chemical doping process introducing the parent probe ^111^In into the samples, or other factors such as the quality and stoichiometry of the samples that allow for the distribution of In\Cd on both lattice sites, which is somehow hindered in our work where ^111^In and ^111*m*^Cd have been introduced by ion implantation or by diffusion. In the particular case of SmCrO_3_ a detailed EFG study as a function of temperature was performed, where ${{\rm{EFG}}}^{{{\rm{R}}}_{1}}$ = ${{\rm{EFG}}}^{{{\rm{Sm}}}_{1}}$ and, the second EFG, ${{\rm{EFG}}}^{{{\rm{Sm}}}_{2}}$, appearing at temperatures below room temperature can only be associated with modifications to the Sm/probe local environments.

### EFG parameters thermal evolution

**Figure 4:**
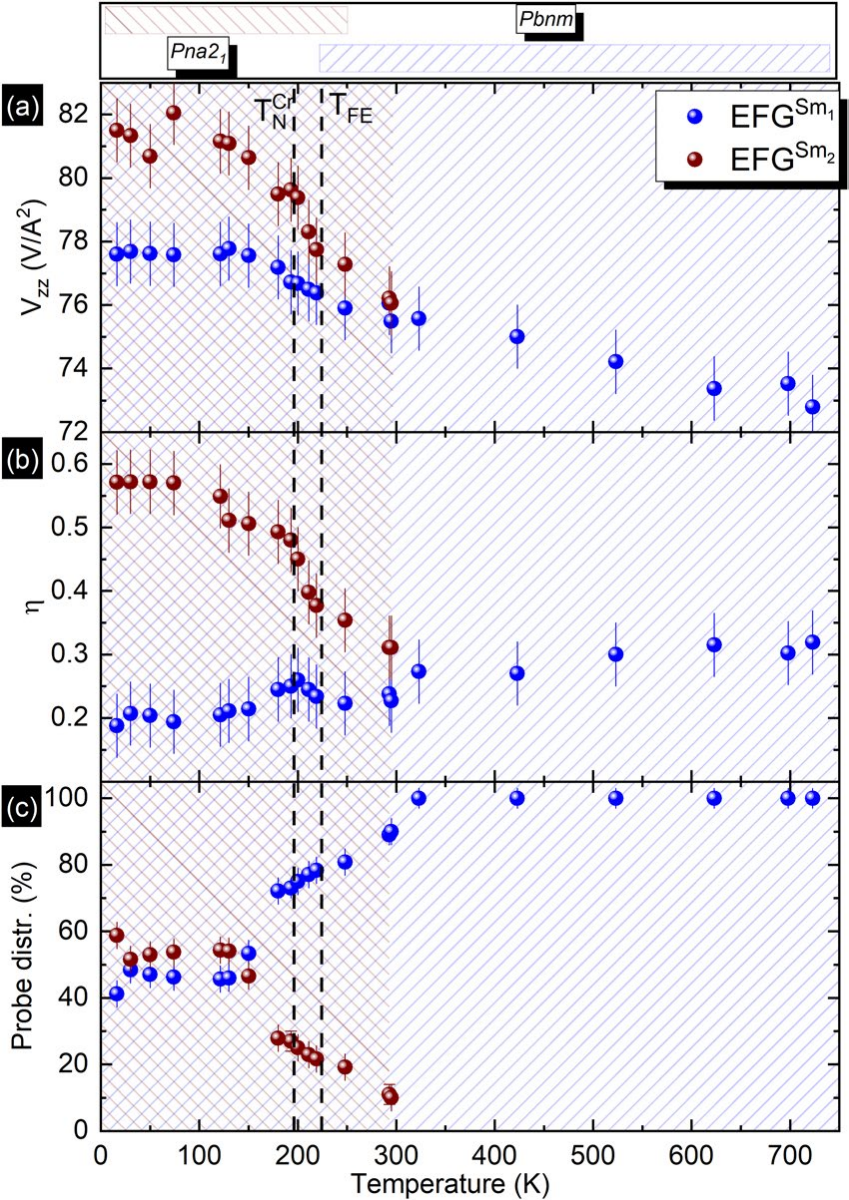
(**a**) Experimental electric field gradient principal component with ^111^In for the SmCrO_3_ sample. (**b**) Asymmetry parameter. (**c**) Probe distribution.

 Figure 4 depicts the thermal dependence of the main parameters of the EFGs for SmCrO3 obtained by our fits. As mentioned, below 300 K, 2 EFGs (${V}_{zz}^{{{\rm{Sm}}}_{1},{{\rm{Sm}}}_{2}}$ and ${\eta }^{{{\rm{Sm}}}_{1},{{\rm{Sm}}}_{2}}$) are observed. Looking in more detail to the ${{\rm{EFG}}}^{{{\rm{Sm}}}_{1}}$ and ${{\rm{EFG}}}^{{{\rm{Sm}}}_{2}}$, one realises that the most relevant difference is the axial asymmetry parameter: while ${{\rm{EFG}}}^{{{\rm{Sm}}}_{1}}$ has ${\eta }^{{{\rm{Sm}}}_{1}}\approx 0.21(5)$, characteristic of a slightly distorted axially symmetric environment, the corresponding ${\eta }^{{{\rm{Sm}}}_{2}}\approx 0.56(5)$ of ${{\rm{EFG}}}^{{{\rm{Sm}}}_{2}}$ reveals a strongly axially asymmetric local environment.

The EFG principal component of both ${{\rm{EFG}}}^{{{\rm{Sm}}}_{1}}$ and ${{\rm{EFG}}}^{{{\rm{Sm}}}_{2}}$ slightly increase as the temperature decreases until ~ 150 K, this increase slows down at lower temperatures. In contrast, while the asymmetry parameter of ${{\rm{EFG}}}^{{{\rm{Sm}}}_{1}}$, ${\eta }^{{{\rm{Sm}}}_{1}}$, slowly decreases upon lowering the temperature for the entire measured range, the asymmetry parameter of ${{\rm{EFG}}}^{{{\rm{Sm}}}_{2}}$, ${\eta }^{{{\rm{Sm}}}_{2}}$, increases significantly below 300 K, down to the lowest measured temperature. One should also mention that the widths of the EFG distributions are nearly temperature independent (${\delta }^{{{\rm{Sm}}}_{1}}\approx 2{\rm{Mrad/s}}\,$ and ${\delta }^{{{\rm{Sm}}}_{2}}\approx 3{\rm{Mrad/s}}\,$). In Fig. 4(c) is also shown the temperature evolution of the fraction of probes interacting with each EFG, *i.e*., the relative abundance of each local environment (LE): ${f}^{{{\rm{Sm}}}_{1}}$ + ${f}^{{{\rm{Sm}}}_{2}}$ = 100%. As pointed, at high temperatures all the probes interact with a single ${{\rm{EFG}}}^{{{\rm{Sm}}}_{1}}$. Upon decreasing the temperature, below 300 K, a second EFG, ${{\rm{EFG}}}^{{{\rm{Sm}}}_{2}}$, is clearly identified for a fraction of probes. Below that temperature ${f}^{S{m}_{1}}$ decreases sharply until 150 K with approximately ${f}^{{{\rm{Sm}}}_{1}}\approx {f}^{{{\rm{Sm}}}_{2}}\approx 50(5) \%$.

**Figure 5:**
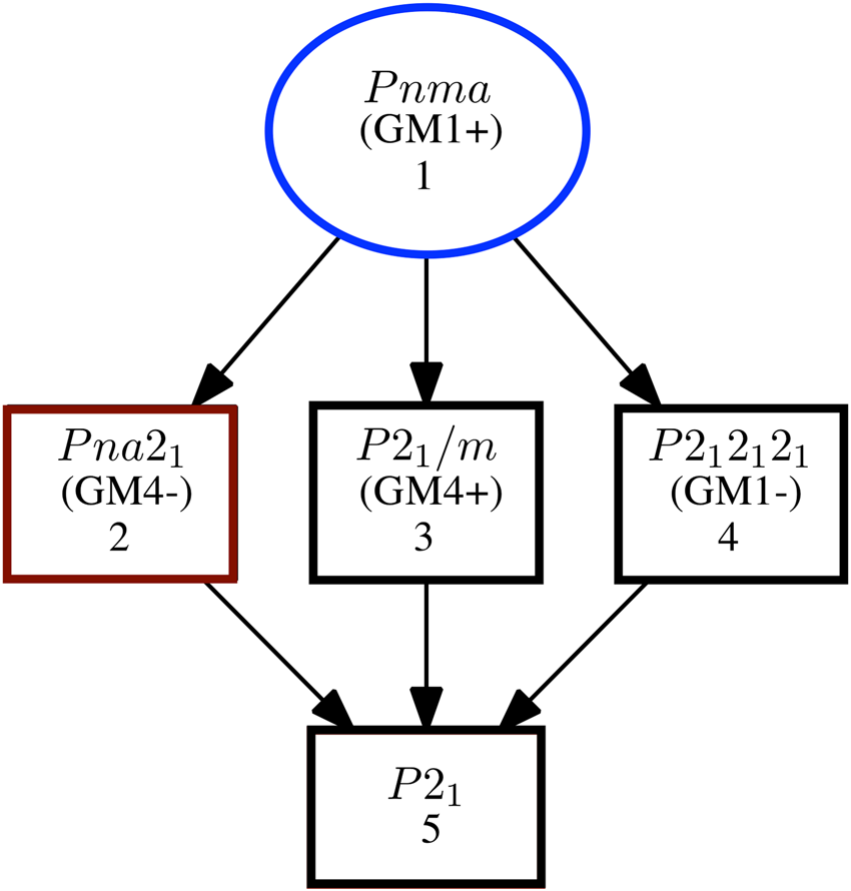
Possible structural phase transitions sequence for *Pnma* space group.

The appearance of a second local environment, approximately emergent below room temperature, determines split fractions of probe atoms sensing a local scale inhomogeneous state in the material. Sm_1_ regular and Sm_2_ distorted environments coexist, due to local distortions probably originated by polar octahedral rotations and/or cation displacements. More unlikely scenarios could be envisaged justifying the appearance of a second EFG considering that at temperatures below 300 K the probe atoms were changing their location via (low temperature) solid state diffusion. Also, the observation of, so-called, “after effects”, which are due to the presence of differently charged probe atoms occurring after the decay of ^111^In to ^111^Cd, and before equilibrium is achieved, can lead to the observation of different EFGs. However, the typical PAC signals due to such processes have a characteristic temperature dependence that is not observed in the present cases. In fact, local distortions have been theoretically predicted, and experimentally observed, below the temperature where polar oxygen octahedral rotations and Sm displacement are suggested (*T* < 240 K)^22^. *Ghosh et al*. relates the emergence of polar order in a paramagnetic state as an indication that ferroloectricity arises from a structural distortion^22^. The authors used a combination of computational software (AMPLIMODE^54^ and ISODISTORT^55^ codes) to search for possible non-centrosymmetric space sub-group of the distorted orthorhombic structure, arriving at a distorted low-T polar *Pna*2_1_ phase. A similar reasoning was used to determine the GdCrO_3_ low temperature *Pna*2_1_ symmetry^24,25^. Taking this into consideration the authors fitted x-ray powder diffraction patterns using a *Pnma* space group at higher temperatures and a *Pna*2_1_ space group at lower temperatures (from all non-centrosymmetric structures studied, *Pna*2_1_ space group better fitted the results). A coexistence of both the structural phases was observed in the range 220–240 K. The authors symmetry analysis revealed that the distortion of *Pna*2_1_ could be decomposed into two modes corresponding to the irreducible representations of non-polar *GM*1+ and polar *GM*4− (see Fig. 5). *Ghosh et al*. also state that a proper ferroelectric transition cannot be described by the current transformation^22^.

### Symmetry assignment

Considering the fact that sometimes it is a laborious and complex task resolving the space group from normal x-ray diffraction spectra, especially in lower symmetry space groups where the use of many refinement parameters is required, we took the present study further and calculated the EFG parameters, using DFT, for two non-centrosymmetric space groups, *Pna*2_1_ and also for an even lower symmetry space group *P*2_1_ [(*Pnma*(N°62) → *Pna*2_1_(N.33) → *P*2_1_(N°4) see Fig. 5] in similarity with what happens in the case of biferroic YCrO_3_^13^. Calculations for the low symmetry centrosymmetric space groups were not performed. Here a SmCrO_3_ structure in the non-centrosymmetric *P*2_1_ space group was created by ISODISTORT, by initially considering the *Pna*2_1_ space group lattice parameter followed by forces minimisation as described before.

The calculated EFG parameters for the Cr and Sm positions considering the previous space groups are summarised in Table 4.

**Table 4:** EFG parameters calculated considering different space groups for the Sm and Cr positions in the SmCrO_3_ perovskite like structure for different space groups.

	Sm		Cr	
	*V*_*zz*_	*η*	*V*_*zz*_	*η*
*Pnma*	52.0(3)	0.03	26.2(3)	0.77
*Pna*2_1_	56.0(3)	0.47	32.1(3)	0.95
*P*2_1_	76.6(3)	0.40	38.6(3)	0.47
	71.0(3)	0.48	21.6(3)	0.22

The EFG parameters obtained for the different low symmetry space groups (see Table 4) show that an increase in the *V*_*zz*_ value and asymmetry parameter for the Sm positions should be expected. Looking into detail, in *P*2_1_ space group we have two non equivalent Sm, that agree with our observation of the two EFG split, *i.e*. two different local environments coexisting at low temperature, below room temperature. However, our calculations predict, for both Sm non-equivalent sites, similar asymmetry parameters near 0.44(4), that is not what we observe experimentally. Differently, the EFG calculations performed for the *Pna*2_1_ space group at the Sm position show similar *V*_*zz*_ value to the one verified at the high temperature phase (*Pnma* space group) but with a much higher asymmetry parameter (0.47), therefore correlating better with our experimental findings for the second EFG, ${{\rm{EFG}}}^{{{\rm{Sm}}}_{2}}$.

Consequently,we believe that, while part of the system associated with ${{\rm{EFG}}}^{{{\rm{Sm}}}_{1}}$ maintains its local orthorhombic symmetry, the remaining part, associated with ${{\rm{EFG}}}^{{{\rm{Sm}}}_{2}}$, appears as a consequence of a local distortion of the initial phase, with axial symmetry breaking compatible with the *Pna*2_1_ space group. Other local techniques such as pair distribution function (PDF) method, using high-resolution neutron powder diffraction data, could prove valuable in order to corroborate our findings.

Finally, the fits below ${T}_{\,{\rm{N}}}^{{\rm{Cr}}\,}$ have been perfectly performed without the inclusion of typical combined interactions (electric field gradient and magnetic hyperfine field interactions, EFG+MHF). In fact, the PAC studies in the RCrO_3_ show that down to the ordering temperature of R, there is not an observable MHF^50,51,53,56^. Only the *R*(*t*) spectrum at *T* = 16 K, *i.e*. below ${T}_{\,{\rm{N}}}^{{\rm{Sm}}\,}$, was fitted considering combined EFG+MHF interactions. Thus, below ${T}_{\,{\rm{N}}}^{{\rm{Sm}}\,}$ the *R*(*t*) experimental data evidences the presence of weak magnetic local hyperfine fields due to the antiferromagnetic ordering of the Sm magnetic sub-lattice. However, the magnetic interaction is much weaker than the electric quadrupole interaction, leading only to an observable frequency broadening. Consequently, the magnetic hyperfine fields were too small to be differentiated at the two local environments, with their magnitudes assumed to be the same within the fit sensitivity, (${B}_{hf}^{S{m}_{1}}\approx {B}_{hf}^{S{m}_{1}}$). The final *R*(*t*) spectra fit was performed accordingly, with a small value around *B*_*hf*_ = 0.3(1) T and a very small angle between the *B*_*hf*_ and *V*_*zz*_ of 1(1)°, that can be treated as zero within the fit sensitivity.

Summarising, in recent reports on this system, local distortions have been theoretically predicted and experimentally observed where polar oxygen octahedral rotations associated with Sm displacement are proposed to be correlated with the development of polar order below 240 K (still in the paramagnetic state)^22^. Our results point to a more exquisite scenario, where locally an inhomogeneous state emerges above magnetic and ferroelectric macroscopic phase transitions and persists to the lowest measured temperature. From our experimental and EFG *ab-initio* calculations the emergent second EFG, *i.e*., the designated second phase, has a *Pna*2_1_ structure. In this new state regular and distorted environments coexist. This inhomogeneous state has passed so far unnoticed and justifies the controversy on whether this system is a magnetically driven improper ferroelectric or not.

## Conclusions

In this work, SmCrO_3_ local structure was studied through the temperature evolution of the electric field gradient. The experimental results were compared with EFG DFT calculations for the RCrO_3_ family. Our SmCrO_3_ data, below 300 K, is compatible with a scenario where local distortions occur, breaking the axial symmetry and permitting the appearance of a ferroelectric order in this system. Yet this emergent distorted environment coexists, down to the lowest measured temperature, with the regular (non-polar) high temperature phase. Our experimental observation, supported by *ab-initio* calculations, thus, reveals an inhomogeneous SmCrO_3_ system with coexisting polar and non-polar states. These inhomogeneities might be behind the RCrO_3_ ambiguous polar structure and represent a step forward in understanding the ferroelectric behaviour of orthochromites.

## Methods

Polycrystalline samples of SmCrO_3_ were prepared with solid state reactions by mixing stoichiometric quantities of Sm_2_O_3_ and Cr_2_O_3_ that were pressed into a pellet and heated 24 h at 1173 K. Two more grinding and consecutive heating steps were performed, 1473 K (24 hours) and 1573 K (48 hours). Phase purity was confirmed by Rietveld refinement of the x-ray powder diffraction data collected with a Panalytical X’Pert Pro diffractometer and analysed with the Fullprof software package^57^. The magnetic properties were probed by measuring isofield magnetisation curves with a commercial (MPMS Quantum Design) Superconducting Quantum Interference Device (SQUID) magnetometer.

To perform the PAC experiments^58^, and thus study the atomic scale properties of this compound a radioactive probe which decays in a double cascade (emitting two photons, *γ*_1_ and *γ*_2_) is introduced in a sample by implantation, diffusion or neutron activation.

For these experiments we have used a bulk polycrystal. The polycrystals were obtained by several grinding, pelletizing and heat treatments leading to a pellet form (about 1 mm thick with 13 mm diameter) . We then cut the pellets, using a diamond wire, into small cubic-like sample with about ~1 × 1 × 1 mm^3^ that are used in the PAC experiments Individual ~1 mm^3^ bulk samples from the same batch material were implanted with ^111*m*^Cd ions [^111*m*^Cd → ^111^Cd, *t*_1/2_ = 49 minutes] with a small dose of 10^11^ atoms/cm^2^ (dose lower than 1ppm of the Sm/Cr concentration) with 30 keV energy at the ISOLDE-CERN facility. The probe beam with 30 keV energy implants into SmCrO_3_ into a depth range of about 10 nm below surface with straggling of 4.3 nm as estimated using SRIM^59^. Complementary studies with ^111^In ions [^111^In → ^111^Cd, *t*_1/2_ = 2.8 days] were performed, but in this case the probes were incorporated in the sample by diffusion process at the Faculty of Sciences of the University of Lisbon. Both ^111^In and ^111*m*^Cd decay to the same PAC probe nuclear excited state with *I* = 5/2 and *Q* = 0.77(12).

A thermal annealing was performed on all samples, to recover from remaining point defects due to the Cd implantation (20 minutes 973 K in air) or to promote In diffusion (48 h 1273 K in air). The recovery of point defects and the incorporation of the Cd probe at the right lattice sites were certified by the PAC measurement itself, leading to well defined *R*(*t*) experimental anisotropy functions, the characteristic PAC observable.

Each temperature measurement took ~3 h acquisition time using a 6-BaF_2_ detector spectrometer^60^ or in a 4-BaF_2_ detector spectrometer at FCUL, equipped with dedicated closed cycle refrigerator or with a special high temperature furnace. Low temperature measurements were done with the sample in vacuum; above room temperature the measurements were done with the sample in air. For further experimental details see, e.g., refs. ^42,60–63^.

Hyperfine interactions, *i.e*., the interactions between the nuclear moments and the extranuclear electromagnetic fields can be measured by several nuclear hyperfine techniques. The measurement of these interactions provides a very sensitive and accurate method to investigate condensed matter phenomena in a large variety of materials, providing direct information on the local charge distribution and magnetic hyperfine fields. Nuclear magnetic (quadrupolar) resonance, muon-spin rotation, nuclear orientation and perturbed angular correlation, are examples of such techniques.

In a solid the nuclei are not isolated and interact with the local environment. If the nuclear moments of a probe atom are known the extranuclear electromagnetic fields can be determined via hyperfine measurements. For example, an asymmetric charge distribution creates an electric field gradient (EFG) which couples with the nuclear electric quadrupole moment (*Q*) of a given probe atom, splitting the nuclear levels. This energy splitting can be measured providing local information.

In PAC spectroscopy, one uses probe nuclei that decay via a two gamma ray radioactive cascade to obtain this local information.

The hyperfine interaction at the probe’s site with the electric quadrupole moment of the intermediate level of the radiactive cascade causes a perturbation in the angular dependence of the emission probability of *γ*_2_ with respect to *γ*_1_ (see Fig. S1). The EFG, characterising the external charge density that interacts with the probe nucleus, is described by a second-order traceless symmetric tensor.

Since the EFG is a traceless matrix and diagonal in its principal axis, it can be completely described by only two parameters: the *V*_*zz*_ component and the axial asymmetry parameter *η* = (*V*_*xx*_ − *V*_*yy*_)/*V*_*zz*_, considering that ∣*V*_*zz*_∣ ≥ ∣*V*_*yy*_∣ ≥ ∣*V*_*xx*_∣. The time-dependent oscillations in the anisotropic emission of *γ*_2_ then define the observable frequency *ω*_0_:

1 $${\omega }_{0}=k\frac{eQ{V}_{zz}}{4I(2I-1)\hslash },$$

where *I* and *Q* are the spin and the electric quadrupole moment of the intermediate level of the cascade, respectively, and *k* = 3 (or 6) for integer (or half-integer) spin. The observable *ω*_*n*_ frequencies relate to the energy splitting of the hyperfine levels created when a nuclear state interacts with the external EFG. For an intermediate level with *I* = 5/2 the quadrupole interaction splits this level into three doubly degenerate sublevels, which is the reason why there is a triplet of frequencies (*ω*_1_, *ω*_2_, *ω*_3_ = *ω*_1_ + *ω*_2_) for each non-vanishing EFG distribution that is observable in the experimental *R*(*t*) spectra and the corresponding Fourier transforms. In the particular case of *η* = 0, the fundamental quadrupolar frequency matches the lower observable, i.e., *ω*_1_ = *ω*_0_, with *ω*_2_ = 2*ω*_0_ and *ω*_3_ = 3*ω*_0_.

The experimental *R*(*t*) function, for a static electric quadrupole interactions can be described as a sum of periodic terms by *R*(*t*) = ∑*A*_*kk*_*G*_*kk*_(*t*), where *A*_*kk*_ are the angular correlation coefficients of the nuclear decay cascade and *G*_*kk*_ contains the perturbation terms of the angular correlation with the relevant information regarding the EFG parameters.

To account for the fact that probes in equivalent sites might have slight deviations in their EFGs, a Lorentzian distribution characterised by their central frequency *ω*_0_ and full width at half maximum (FWHM) is integrated for each EFG in the perturbation function, *i.e*., when in the presence of EFG distributions the periodic terms are attenuated, and thus the perturbation function can be described as:

2 $${G}_{kk}(t)={S}_{k0}+\sum _{n}{S}_{kn}\cos ({\omega }_{n}t){e}^{-\delta {\omega }_{n}t}.$$

The PAC experimental observable, *R*(*t*) anisotropy function, was fitted with exact numerical methods that build the expected observable by solving the characteristic equations of the hyperfine interaction Hamiltonian^58,61^. Experimentally, the coincidence spectra *N*(*θ*, *t*) are recorded, where *θ* is the angle between detectors and *t* is the time delay between the detection of *γ*_1_ and *γ*_2_, allowing for the experimental perturbation function *R*(*t*) to be calculated:

3 $$R(t)=2\frac{N(18{0}^{\circ },t)-N(9{0}^{\circ },t)}{N(18{0}^{\circ },t)+2N(9{0}^{\circ },t)}.$$

Each *N*(*θ*, *t*) is represented by a sum proportional to the perturbation factor:

4 $${G}_{{k}_{1}{k}_{2}}^{{N}_{1}{N}_{2}}(t)=\sum _{n}{S}_{{k}_{1}{k}_{2},n}^{{N}_{1}{N}_{2}}\cos (\omega t).$$

## Supplementary information

Supplementary Information.
