# Supplementary material for: Local inhomogeneous state in multiferroic SmCrO3
Source: Sci Rep. 2020 Mar 13;10:4686. doi: 10.1038/s41598-020-61384-6 (PMC7070006; doi:10.1038/s41598-020-61384-6)
Supplement: Supplementary file 1 — Supplementary Information. [file 41598_2020_61384_MOESM1_ESM.pdf]

Local inhomogeneous state in multiferroic $SmCrO_3$

G. N. P. Oliveira[1,*], R. C. Teixeira[1], R. P. Moreira[1], J. G. Correia[2], J. P. Arau´jo[1], and A. M. L. Lopes[1,+]

[1]IFIMUP -Instituto de F´ısica de Materiais Avançados, Nanotecnologia e Fotónica, Departamento de Física e Astronomia da Faculdade de Ciências da Universidade do Porto, Rua do Campo Alegre, 687, 4169-007 Porto, Portugal

[2]C2TN, Centro de Ciências e Tecnologias Nucleares, Departamento de Engenharia e Ciências Nucleares, Instituto Superior Técnico, Universidade de Lisboa, Estrada Nacional 10, 2695-066 Bobadela LRS, Portugal

[*, +] Correspondence and requests for materials should be addressed to:

goliveira@fc.up.pt; armandina.lima.lopes@cern.ch

S.1 Perturbed angular correlations sketch

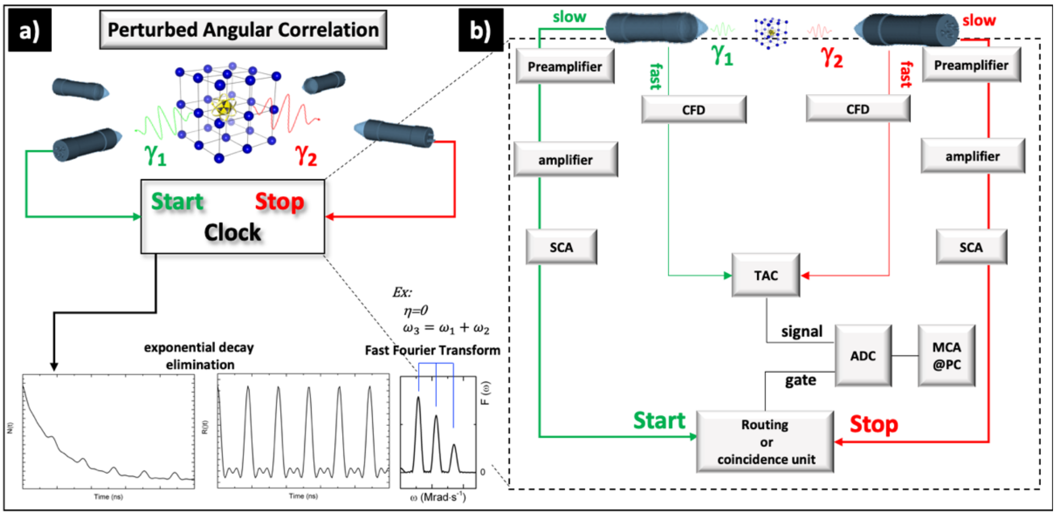

Figure S1. Basic representation of the gamma-gamma (Time Dependent) PAC technique: **a)** events-count histogram measured as a function of time between the emission of correlated gammas from the decay cascade. With these exponential decay spectra taken at 180°and 90°detector angles the exponential term is eliminated with the construction of the “anisotropy ratio” experimental function R(t). R(t) evidences only the perturbation function due to the hyperfine interactions resulting from the interaction of the nuclear moments of the intermediate cascade state with the external EFG (and B) fields. **b)** Detailed view of the “clock” mechanism, *i.e.*, slow-fast electronics used to analyse the gamma-ray energies, determine their time interval with sub ns precision, and build the coincidences histograms for all detector’s combinations pairs at 180°and 90°angles.
